# Handling missing data: AI approach for survival prediction in lung cancer despite missing data

**DOI:** 10.1038/s41746-026-03019-0

**Published:** 2026-07-16

**Authors:** Margaret Y. Sui, Kyra L. Rosen, Ariel Yuhan Ong, Joseph C. Kvedar

**Affiliations:** 1 Harvard Medical School, Boston, MA USA; 2 Institute of Ophthalmology, University College London, London, UK; 3 Moorfields Eye Hospital NHS Foundation Trust, London, UK; 4 NIHR Moorfields Biomedical Research Centre, London, UK

**Keywords:** Cancer, Computational biology and bioinformatics, Medical research, Oncology

## Abstract

Optimal cancer prognostication combines multimodal data including biopsy results, CT scan, patient characteristics, and clinical trajectory thus far. However, many patients do not have all modalities of data available, so requiring physicians to have all patient data modalities to use an AI prediction algorithm limits the tool’s clinical utility. To increase the clinical potential of these AI algorithms, Ruffini et al. developed a “missing data aware” survival prediction approach that is able to handle inputs with missing data for risk stratification in non-small cell lung cancer patients.

## Introduction

For many cancer patients, prognostication is the most important question on their minds when they are diagnosed. Cancer prognostication and stratifying patients into high versus low risk buckets for adverse events like metastasis helps physicians make treatment and surveillance decisions. All cancer treatments have negative side effects such as immunocompromise and severe nausea, so while maximum likelihood of cancer eradication is the goal for many patients, predicted patient survival informs the type and quantity of treatments that it makes sense to offer.

Despite increasing research on specific cancer markers that can inform cancer aggression, prognostication remains challenging, and many physicians hesitate to give patients an exact answer. In one study of nearly 600 patients with advanced cancer, only 17.6% of the 71% who wanted to know their prognosis were given one, yet patients who had a better estimate of their prognosis were less likely to pursue futile treatment options that lowered their quality of life^1^. Even for palliative care physicians who do give a prognosis, one study estimated that only 41% of those prognoses were actually accurate, and out of the inaccurate estimates, 85% were overestimates^2^. This gap leaves room for more objective prognosis estimators.

Modalities of data that are used these days for determining whether a cancer is high or low risk for recurrence and metastasis include biopsy data, imaging, cancer genomic information, and patient clinical characteristics^3–5^. Given the different advantages that various data modalities provide for cancer risk stratification, many successful AI approaches these days rely on “multi-modal” inputs. However, for non-small cell lung cancer, realistically only ⅓ patients are biopsied due to risks like lung collapse^6^. Decisions to biopsy depend on the location of the tumor, its clinical utility for decision making (i.e., whether it is already known from CT scan that it is an aggressive tumor), and whether the patient would be able to tolerate the procedure. Both CT scans and clinical data are more commonly available for patients; however, not everyone receives a CT scan and information on relevant genes that impact cancer growth and other medical history might be missing from the patients’ chart. In Ruffini et al.’s study, out of 179 patients, only one-fifth had all available data modalities complete^6^. To address this challenge of incomplete data, Ruffini et al. developed an approach to use AI to learn patient risk even when some modalities of data are missing out of multi-modal histopathology, CT scan, and clinical courses from the electronic health record (EHR)^6^.

## Approach

As the first step to their algorithm, Ruffini et al. used pretrained foundational models to extract important features from each of the individual histopathology, CT scan, and clinical course data modalities available^7–13^. After extracting these features, they performed modality encoding that translates the raw data from each modality into a numerical vector. For this modality encoding, they used the Not Another Imputation Method (NAIM)^14^, which handles missing data without first needing to impute, or guess, the missing inputs^14^, followed by an Oblivious Differentiable Sparsemax Trees (ODST) encoder^15^ which can handle non-linear, higher-order interactions between features^15^. The vectors output from the NAIM + ODST encoder for each modality are then concatenated into one large vector to be used as the input for the final risk prediction.

The authors tested three data modality fusion strategies: early, intermediate, and late fusion. Early fusion involves fusing the data together at the starting feature level, intermediate fusion involves fusing the data together after performing modality specific encoding (such as described in the paragraph above), and late fusion involves obtaining separate risk prediction for individual modalities and then taking a weighted risk. The authors tested all three approaches and found that intermediate fusion generally provided the most accurate risk assessment. Compared to a unimodal concordance index (C-index) baseline of 59–69%, this intermediate fusion approach yielded a C-index of 74 ± 4%. The C-index measures the probability that given two randomly selected patients in the dataset, the one who experienced an earlier adverse event had a higher predicted risk^6^. Thus, the higher the C-index, the better the prediction, and a C-index of 50% represents predictions no better than a coin flip. Early fusion yielded a C-index of 73 ± 4%, intermediate fusion yielded a C-index of 74 ± 4%, and late fusion yielded a C-index of 72 ± 5%. Intermediate fusion yielded a very marginal improvement over these other two strategies, but all strategies performed comparably.

To prevent overfitting on their data, which was a small dataset with only 179 patients with stage II-III non-small cell lung cancer, the authors performed 5-fold cross validation, which involves training on four-fifth of the patients and leaving out one-fifth of the patients’ data to use for evaluating the model. In addition to reporting the C-index, the authors also reported the time-dependent AUC (td-AUC) and Uno’s C-index. For the intermediate fusion, the td-AUC was 81 ± 5% compared to a unimodal baseline of 55–75%, and the Uno’s C-index was 69 ± 4% compared to a unimodal baseline of 56–64%. Overall, combining data enabled a modest improvement in prediction accuracy compared to analyses performed on one data modality at a time.

## Clinical utility and limitations

In medical practice, risk prediction tools like this multi-modal artificial intelligence (AI) algorithm could help provide physicians with an important prognosis metric to guide treatment planning and allow more patients to receive the prognosis they seek, delivered with appropriate confidence bounds. While there are concrete known prognosticators that physicians can look for in patient data to provide a comprehensive view on how aggressively to surveil and treat the cancer, there are also many unknown prognosticators hidden in the data that AI might be able to pick up on and use to further inform risk^5^. And given the challenges of accurate physician prognoses and human bias that can affect the prognoses that physicians want to give patients, such tools might help provide more objective metrics as guidelines.

Tools like these AI algorithms that take into account large amounts of patients’ individual data when performing prognostication are now becoming increasingly accurate in research settings where the testing is performed on similar data to the training data. However, hurdles remain in the tool’s generalizability across different hospitals and settings^16,17^. Furthermore, since patient data is being used and the accuracy of these tools is crucial for treatment planning, regulation of these tools is critical prior to clinical use. Currently, regulatory hurdles remain challenging for many AI tools to overcome.

Another challenge to such tools is that physicians may easily put too much or too little trust into the accuracy of such a tool for their clinical setting. An inaccurate prognostic prediction could easily negatively affect treatment planning, and inaccurate predictions are more likely for patients with rarer presentations since these tools rely on picking up patterns from the training data. Rarer cases are also where tools like this would be most critical because it is more difficult to make clinical decisions when there is less literature available and less existing clinical experience to draw from. In non-small cell lung cancers, rare subtypes such as large cell neuroendocrine carcinoma, adenosquamous carcinoma, and sarcomatoid carcinoma are aggressive subtypes precisely where prognostic guidance is most needed^18–20^. It is important for these AI tools to address rare presentations carefully, and both patients and physicians should keep in mind that the risk prediction from these AI tools provides a single imperfect estimate rather than serving as truth.

## Conclusion

To make AI tools and approaches more applicable in medicine, these tools would need to both integrate information from a large number of data modalities and handle missing data modalities when necessary. Ruffini et al. have tackled this challenge by designing an AI algorithm that first processes each available modality separately, then transforms available data modalities into a combinable form, and finally renders a risk prediction based on the combined data. While still less able to flexibly consider various available data modalities than physicians, these algorithms are a first step towards serving as an additional input to clinical judgment that is more flexible towards variable inputs than prior AI models. In the future, the predictions that these AI algorithms make could potentially guide medical decision-making and help patients and physicians make personalized, risk-informed decisions.

## Data Availability

No datasets were generated or analyzed during the current study.
